# A549 Cell-Covered Electrodes as a Sensing Element for Detection of Effects of Zn^2+^ Ions in a Solution

**DOI:** 10.3390/nano12193493

**Published:** 2022-10-06

**Authors:** Mina Eghbal, Martin Rozman, Veno Kononenko, Matej Hočevar, Damjana Drobne

**Affiliations:** 1 Department of Biology, Biotechnical Faculty, University of Ljubljana, Večna pot 111, 1000 Ljubljana, Slovenia; 2 FunGlass—Centre for Functional and Surface Functionalized Glass, Alexander Dubček University of Trenčín, Študentská 2, 911 50 Trenčín, Slovakia; 3 Institute of Metals and Technology, Lepi pot 11, 1000 Ljubljana, Slovenia

**Keywords:** electrochemical cell-based impedance spectroscopy (ECIS), A549 cells, stainless steel, ZnCl_2_, cytotoxicity, biosensor, detector

## Abstract

Electrochemical-based biosensors have the potential to be a fast, label-free, simple approach to detecting the effects of cytotoxic substances in liquid media. In the work presented here, a cell-based electrochemical biosensor was developed and evaluated to detect the cytotoxic effects of Zn^2+^ ions in a solution as a reference test chemical. A549 cells were attached to the surface of stainless-steel electrodes. After treatment with ZnCl_2_, the morphological changes of the cells and, ultimately, their death and detachment from the electrode surface as cytotoxic effects were detected through changes in the electrical signal. Electrochemical cell-based impedance spectroscopy (ECIS) measurements were conducted with cytotoxicity tests and microscopic observation to investigate the behavior of the A549 cells. As expected, the Zn^2+^ ions caused changes in cell confluency and spreading, which were checked by light microscopy, while the cell morphology and attachment pattern were explored by scanning electron microscopy (SEM). The ECIS measurements confirmed the ability of the biosensor to detect the effects of Zn^2+^ ions on A549 cells attached to the low-cost stainless-steel surfaces and its potential for use as an inexpensive detector for a broad range of chemicals and nanomaterials in their cytotoxic concentrations.

## 1. Introduction

In recent years, the obtainment of easily accessible, accurate, real-time data on the effects of different chemicals as pollutants has become a crucial environmental issue drawing great public concern and scientific attention. Therefore, there is a growing interest in developing label-free, low-cost, easy-to-use methods that provide qualitative and quantitative on-site information about the effects of chemicals (pollutants) without the need for sophisticated instruments that require extended preparation of samples [1,2,3]. Colorimetric and electrochemical biosensors present a cost-effective alternative to conventional toxicity tests [4], especially in places where ease of use and cost are important. There are also methods that tend to combine the simplicity of the measurement, without lengthy preparation, with high accuracy. One such example is the impedance cell-based spectroscopy method, which is already known in the area of cytotoxicity measurement [5,6]. These methods may provide a label-free, on-site technique that can electrochemically identify and quantify the effects of a variety of different compounds or their mixtures. The cellular morphological responses to the compound reflect the effects of analysts and provide physiologically and biologically more relevant information [6,7,8,9,10]. Electric cell-substrate impedance sensing (ECIS) uses high-end potentiostats/galvanostats with disposable liquid holders that have electrodes attached and on which the living cells grow. These are now commercially available systems that have been developed and applied to different aspects of the cytotoxicity field [11,12,13,14]. It was observed that the cytotoxic effects evaluated by impedance-based systems were in good agreement with standard cytotoxicity methods [15], where ECIS can also detect cellular changes, such as cell detachment, morphological changes during apoptosis, cell spreading, and proliferation [16,17,18,19], in a quantitative and non-invasive manner [20,21,22]. Currently available cell-based commercial platforms, such as xCELLigence [23], can provide accurate, real-time, label-free analysis; however, its use of large equipment and disposable gold microelectrodes makes it less convenient for large-scale field analysis [24].

In our study, stainless steel (SS) was used as an electrode material instead of gold to reduce the costs of the electrodes. Steel foil is a commercially easily accessible, available, inexpensive material [25] that has been previously used as an electrode material with great success in biosensors [26]. Specialized types of steel are used in many biomedical tools and devices as steel possesses unique characteristics, including satisfactory electrical conductivity, high mechanical strength, and acceptable biocompatibility [27]. As such, portable devices for cytotoxicity detection using inexpensive electrodes with good characteristics may present a promising approach for fast and inexpensive cytotoxicity investigations. 

In the study presented here, A549 human lung adenocarcinoma cells were used as a biological sensing element due to their good characteristics [28], such as their ability to form a homogeneous confluent monolayer on different surfaces [29] and their ability to indicate good correlations between ECIS signal changes and the cellular morphology. As a response to stress, A549 cells change morphology, spreading pattern, and adhesion when exposed to a harsh environment, which can provide relevant information on stressful conditions.

The main motivation for this work was to develop an easy, cheap, on-site quantitative method to detect the cytotoxic effect of studied substances (pollutants) and their mixtures in the liquid environment. The cell adhesion to the steel surface and their morphological changes after treatment with a selected pollutant (Zn^2+^ ions) were monitored in detail by light and scanning electron microscopy (SEM). The SEM was used to examine the cell shapes, protrusions, and cell surface morphologies of differently treated A549 cells. Zinc ions were chosen as a representative metal pollutant and stress inducer [30,31,32]. It was also observed that Zn^2+^ ions have a concentration-dependent cytotoxic effect [33].

**Scheme 1:**
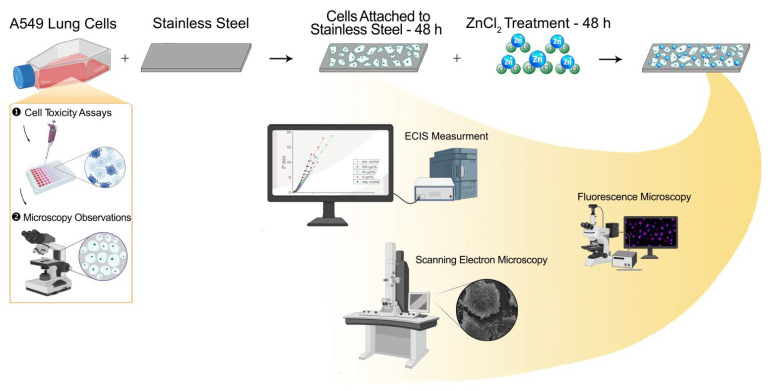
Visual representation of work presented in this manuscript; presenting cell adhesion on the electrodes, treatment of the samples, and methods used.

The assembled biosensor uses three-electrode configurations with electrochemical cell-based impedance spectroscopy (ECIS) as the main mode of detection. In parallel, the effect of the Zn^2+^ ions on cell morphology (cell spreading, detachment, and death) was investigated using light and electron microscopy to confirm and validate the ECIS measurement (Scheme 1). The presented work discusses the possibility of adhering A549 cells onto an inexpensive substrate in order to detect any hazardous potential of the substances or their mixtures in a liquid medium.

## 2. Materials and Methods

### 2.1. Chemicals and Reagents

Coomassie Blue dye, Na-phosphate buffer, and paraformaldehyde were purchased from Merck (Steinheim, Germany). Karnovsky’s fixative, OsO_4_, and hexamethyldisiloxane (HMDSO) were bought from SPI Supplies (West Chester, PA, USA). Milli-Q water was used to prepare solutions. Stainless-steel foil (50 μm) AISI 321 was obtained from Goodfellow (Hamburg, Germany). Carbon HB Pencil was bought from Staedtler (Nuremberg, Germany). All other chemicals and reagents were purchased from Sigma-Aldrich (Munich, Germany) unless otherwise mentioned.

### 2.2. Cell Culture

Lung cancer cells (A549 cells) were obtained from the ATCC cell bank. For all experiments, cells with passage numbers 50–60 were used. A549 cells were cultured in Dulbecco’s modified Eagle’s medium (DMEM), supplemented with 2 mM L-glutamine and 10% (*v/v*) FBS at 37 °C in a humidified atmosphere with 5% CO_2,_ and were routinely passaged once a week.

### 2.3. Cytotoxicity Tests

For all cytotoxicity experiments, A549 cells (2.2 × 10^4^ cells/cm^2^) were seeded in 96-well plates. After 24 h incubation to allow the cells to adhere, cells were treated with a serial dilution of ZnCl_2_ (concentrations 10, 40, 80, and 320 µg/mL). After 24 h treatment, the cytotoxicity was measured by resazurin assay and neutral red uptake (NRU) assay, as described previously [34]. For the resazurin assay, 25 µg/mL of resazurin was added to each well and incubated at 37 °C for two h. Fluorescence intensity of formed resorufin was measured (560/590 nm ex/em) using a spectrofluorometer (BioTek, Cytation 3). For each treatment condition, at least three independent experimental repeats, each with three replicates, were performed. For the NRU assay, 0.04 mg/mL neutral red dye was added to each well, and cells were incubated for 2 h, allowing dye to become trapped inside the acid organelles. Cells were rinsed with PBS, followed by release of the internalized dye by a prepared solvent (50% *v/v* ethanol, 1% *v/v* acetic acid, and 49% *v/v* deionized water). Fluorescence of the released neutral red dye was measured spectrofluorimetrically (BioTek, Cytation3) at an excitation wavelength of 530 nm and an emission wavelength of 645 nm. For each treatment condition, at least three independent experimental repeats, each with three replicates, were performed.

### 2.4. Stainless-Steel Electrode Preparation (WE)

#### 2.4.1. Stainless-Steel Cleaning Procedure

Stainless-steel surfaces were exposed to UV light for 30 min and then put in absolute ethanol for 15 min to be sterilized.

#### 2.4.2. Cell Attachment to the Stainless-Steel Surfaces

Cleaned and sterilized stainless-steel surfaces (working electrodes in ECIS) were placed in the center of each well of the 12-well plates. Trypsinized A549 cells suspended in supplemented cell culture medium were plated at a seeding density of 2.2 × 10^4^ cells/cm^2^ into each well with stainless-steel electrodes. Submerged steel surfaces in 12-well plates were left in an incubator for 2 days, so cells could attach to the surfaces and grow.

#### 2.4.3. Cell Treatment

After two days, cells were treated with different concentrations of ZnCl_2_ (10, 40, 80, and 320 µg/mL) for another two days. Untreated cells were used as control and surfaces without cells as blanks. Prepared stainless-steel sensing elements were checked with optical microscopy and analyzed with different microscopy methods and ECIS.

### 2.5. Fluorescence Microscopy

The stainless-steel surfaces prepared as working electrodes (WE) after ZnCl_2_ treatment) were rinsed with PBS and stained with 2 μg/mL Hoechst 33342 and 2 μg/mL propidium iodide for 20 min to stain the nuclei of all cells blue and nuclei of nonviable cells red since PI can pass only through damaged plasma membrane. The number of live cells was obtained by subtracting the number of PI-positive cells from the total number of cells that were stained by Hoechst 33342. Data were obtained from three independent experiments, and five random images were analyzed for each condition. Random images of stained cells on the stainless-steel surface were taken with a fluorescent microscope (Zeiss, Germany). ImageJ was used to quantify the density of attached cells and cell viability.

### 2.6. Scanning Electron Microscopy Imaging

Cell-covered surfaces (WE) were washed with PBS and then fixed with Karnovsky’s fixative over two days at 4 °C. After rinsing samples with Na-phosphate buffer, the post-fixation procedure was performed with 1% OsO_4_ for 1 h followed by 3× dH_2_O washes. Then, saturated thiocarbohydrazide solution was added to samples for 15 min and washed three times with water, and the OsO_4_ step was repeated. To dehydrate the samples, a serial dilution of ethanol and HDMS was used. After 24 h, the fully dried samples were sputtered with Au/Pd (thickness: 6 nm) using Precision Etching Coating System (PECS). SEM (JEOL, Tokyo, Japan) was used to visualize the attachment and morphology of A549 cells on surfaces. Random images were taken from the ZnCl_2_-treated WEs. Data were obtained from 3 independent experiments and 5 random images were analyzed for each condition. ImageJ was used to calculate cell coverage.

### 2.7. Electrochemical Impedance Spectroscopy

#### 2.7.1. Biosensor Construction

The biosensor device was constructed using three electrodes including a working electrode (WE) (as described above), reference electrode (RE), and counter electrode (CE)—all made from stainless steel with modifications. A plain steel electrode served as the RE, which was made from the same material as the WE, with the exclusion of living cells to negate any side reactions during measurement that could be due to corrosion of steel in the buffer media as a result of prolonged exposure. A stainless-steel counter electrode (CE) was prepared by gently scrubbing already cleaned stainless-steel foil with an HB lead pencil until it was fully covered with graphite. The stainless-steel CE was then used in an assembled biosensor device.

The WE, CE, and RE were assembled in a cuvette well, where each individual electrode was covered with insulation tape on the backside, placed in the well, and attached with connecting clips to the connector wire and side of the cuvette. First, the RE and CE were placed facing each other on opposite sides of the cuvette. Then, 1 mL of PBS was added. The cuvette was then fixed in the fixating clamp. Lastly, the WE was carefully inserted into the cuvette and connected with the clip. The device remained undisturbed during measurement.

#### 2.7.2. ECIS Measurement

ECIS was used to detect surface changes on the electrode by using a low-voltage alternating signal applied at different frequencies [35,36]. By performing an ECIS measurement, changes in the electrode surface and electrolyte conductivity can be assessed, which may indicate any changes in cells adhered to the surface (proliferation, cell spreading, and cell adhesion) when exposed to increasing concentrations of chemicals [37].

ECIS was carried out using a potentiostat/galvanostat (Palmsens4, the Netherlands) in a frequency range of 0.1–100 kHz with an AC amplitude of 10 mV with a potential set toward a pre-measured open-circuit potential (OCP) to determine that the assembled devices were operational. The measurement was performed using a three-electrode setup (as mentioned above). Before each measurement, the OCP was calibrated to confirm that the assembled device connection is stable. The WE was replaced between each measurement. A new device was constructed using the new CE and RE for different groups of WE samples (treated with different concentrations of ZnCl_2_) to prevent cross-contamination, which could have an impact on measurements. Concentrations selected for ECIS measurement of the biosensoricsresponse of A549 lung cells were selected at 9, 40, and 200 µg/mL. In addition, each experiment for a separate sample was repeated 3 times.

### 2.8. Statistical Analysis

The data from cytotoxicity and fluorescence experiments were statistically analyzed by unpaired t-test for multiple comparisons. A *p*-value lower than 0.05 was considered statistically significant. All statistical analyses were performed using GraphPad Prism 6 software.

## 3. Results

### 3.1. Cytotoxicity of ZnCl_2_

**Figure 1:**
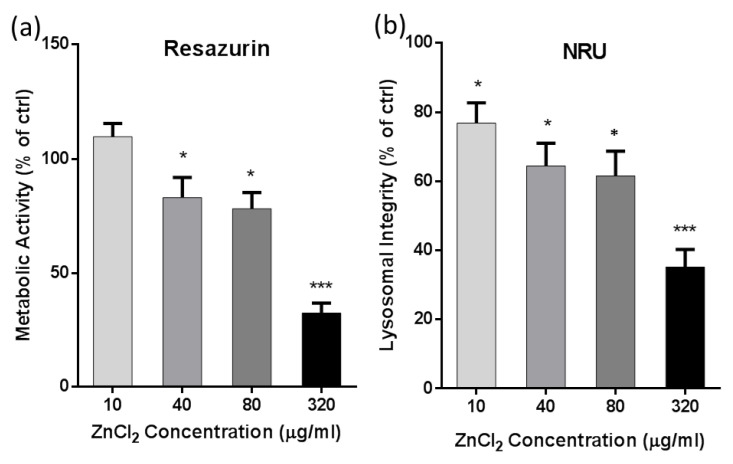
Cytotoxicity of ZnCl_2_ to A549 cells. Cytotoxicity was assessed after 24 h exposure to ZnCl_2_ and evaluated by (**a**) resazurin assay and (**b**) neutral red uptake (NRU) assay. For each treatment condition, at least three independent experimental repeats were performed. Data are presented as the means +SEM of untreated control. Asterisk presents significant difference with respect to the untreated control cells (* equals *p* < 0.05; *** equals *p* < 0.001; unpaired *t*-test).

To determine the toxicity of Zn^2+^ ions on A549 cells, cell metabolic activity and lysosomal integrity were measured by resazurin assay and neutral red uptake (NRU) assay. As shown in Figure 1a, the metabolic activity of the A549 cells exposed to 10 µg/mL of ZnCl_2_ was unaffected, while a significant decrease in metabolic activity was observed in the groups treated with higher ZnCl_2_ concentrations. The metabolic activity of the A549 cells exposed to 320 µg/mL of ZnCl_2_ dropped below 50% in comparison to control cells. The NRU assay detects a small but statistically significant change in the lysosomal integrity of the ZnCl_2_-treated A549 cells already at 10 µg/mL (Figure 1b).

### 3.2. Optical Microscopy Imaging

**Figure 2:**
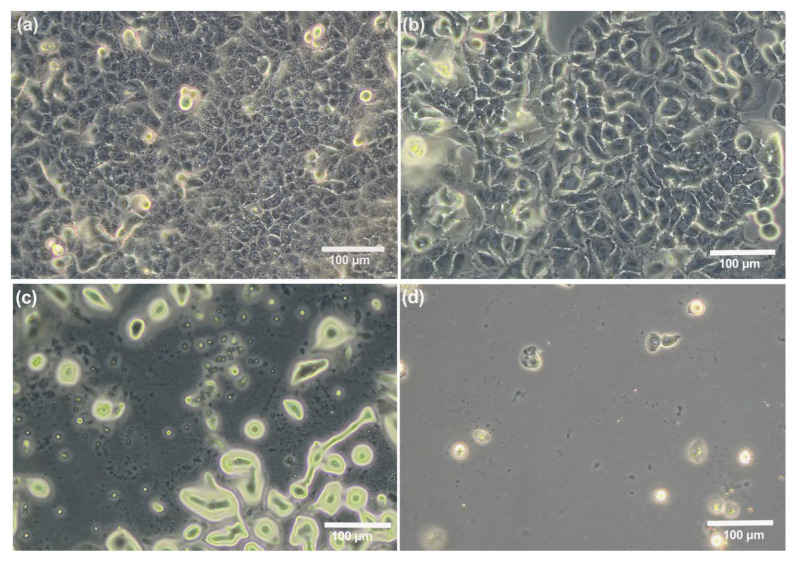
Optical microscopy images of A549 cells seeded in 12-well plates after 48 h treatment with different concentrations of ZnCl_2_. Control cells formed a complete monolayer; cells treated with 10 µg/mL of ZnCl_2_ are still covering the entire surface, but, in higher ZnCl_2_ concentrations, the majority of cells were detached. (**a**) Untreated A549 cells and (**b**–**d**) cells treated with 10, 40, and 320 µg/mL of ZnCl_2,_ respectively.

As depicted in Figure 2a, the untreated A549 cells retained their common shape and tightly covered the entire surface. Furthermore, the fully cell-covered surfaces were treated with ZnCl_2_ for 2 days to figure out the effect of Zn^2+^ ions on the A549 cells seeded on steel surfaces. There is a decreasing trend in the number of cells from the lowest concentration to the highest concentration compared to the control group; the ZnCl_2_-treated cells were detached from the surfaces in a dose-responsive manner. The surface treated with 320 µg/mL ZnCl_2_ was almost without cells after two days, as shown in Figure 2d.

### 3.3. Cell Viability Evaluation

For cell viability evaluation, live/dead fluorescent staining and microscopic observation were applied. Stainless-steel surfaces covered by cells were stained with propidium iodide (PI) and Hoechst 33342 after two days of exposure to ZnCl_2_, and the results were interpreted qualitatively and quantitatively.

**Figure 3:**
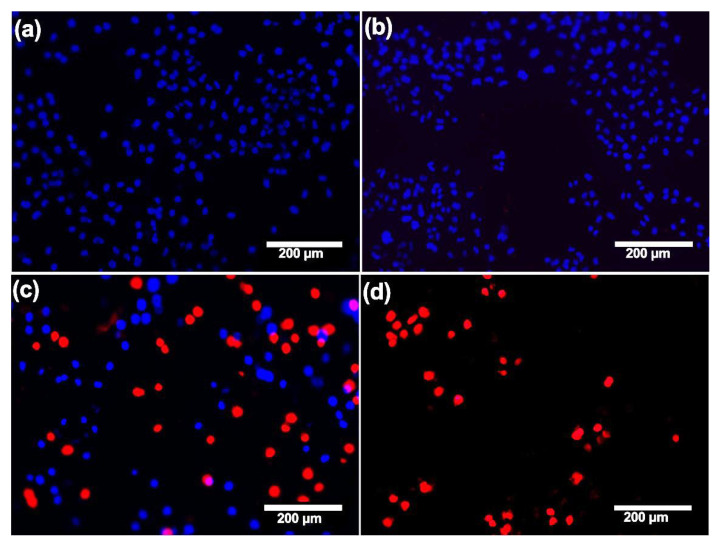
Fluoresce microscopy images of A549 cells on stainless-steel surfaces after 48 h treatment with different concentrations of ZnCl_2_. Cells stained with Hoechst 33342 and PI (nuclei of viable cells are blue, and nuclei of dead cells are red). The number of cells decreases in a dose-dependent manner, and almost all of the remaining cells are dead. (**a**) Untreated cells and cells treated with (**b**) 10, (**c**) 40, and (**d**) 320 µg/mL of ZnCl_2_.

Figure 3 describes how dead and living cells covered the surface. In the lower concentrations, almost all cells covering the surface are live (blue nuclei), while, in the highest concentration, almost all attached cells are dead (red nuclei). The number of attached cells on the control (non-treated A549 sample) is significantly higher in comparison with the number of attached cells on the treated samples after 48 h. The total number of cells present on the surface, whether live or dead, decreased significantly in a dose-dependent manner.

**Figure 4:**
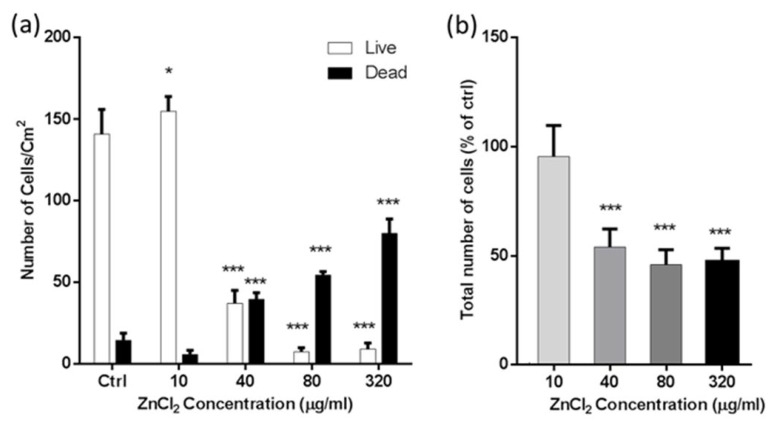
A549 cells adhered to the stainless-steel surface (dead and alive cells) after 48 h treatment with ZnCl_2_. (**a**) Number of dead and live cells in exposure with different concentrations of ZnCl_2_ and (**b**) total number of cells attached to the surface (percentage of control). (Data are presented as the means +SEM versus the control. Asterisk presents significant difference with respect to the untreated control cells (* equals *p* < 0.05; *** equals *p* < 0.001; Unpaired *t*-test).

Figure 4a,b discuss the above-explained data quantitatively. In concentrations of 10 µg/mL of ZnCl_2_, 2 ± 1% of the attached cells were dead, and surfaces were mostly covered with live cells. The number of dead cells increased when the concentration of ZnCl_2_ was increased. At the concentration of 40 µg/mL, more than 55% of cells were already detached from the surface, and the majority of the attached cells were dead. In the concentration of 320 µg/mL, 92 ± 9 of the attached cells were dead. (Figure 4a,b).

Fluorescence microscopy results reveal that a higher concentration of Zn^2+^ ions decreases the total number of attached cells and also increases the number of dead attached cells. Eventually, in the highest concentration, the majority of the cells were detached, and the majority of the remaining ones on the surface were dead.

### 3.4. Shape and Attachment Pattern of Cells

After the preparation of the working electrodes, the treated stainless-steel surfaces with the Zn^2+^ ions were fixated through a fixation procedure and were imaged randomly with the SEM to examine the changes in the cell attachments, surface coverages, and morphologies of the samples. 

**Figure 5:**
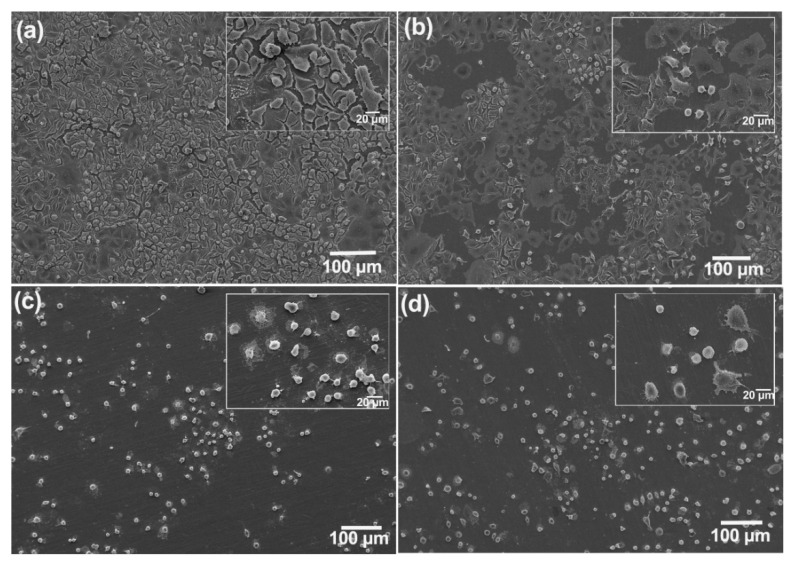
SEM images of A549 cells on stainless-steel surfaces after 48 h treatment with different concentrations of ZnCl_2_. A549 cells covered almost the whole surface in untreated samples (**a**), and cells gradually detached from the surface in a dose-dependent manner; (**b**–**d**) are concentrations: 10, 40, and 320 µg/mL of ZnCl_2_.

The status of viability and behavior of the cells attached to the surface is essential to act as sensing elements. It is important to determine whether the cells are dying or will be dead and detached so that they can change the surface features and be readily detected by ECIS [18]. As shown in Figure 5a, the control (untreated A549 cells) covered the stainless-steel surfaces considerably, whereas a slight decline in the number of attached cells was observed in the samples treated with 10 µg/mL of ZnCl_2_. At the concentration of 40 µg/mL, the number of cells on the surface is greatly decreased. Significant changes in cell morphology can be seen in this concentration. In the highest concentration, almost all cells are detached from the surface.

**Figure 6:**
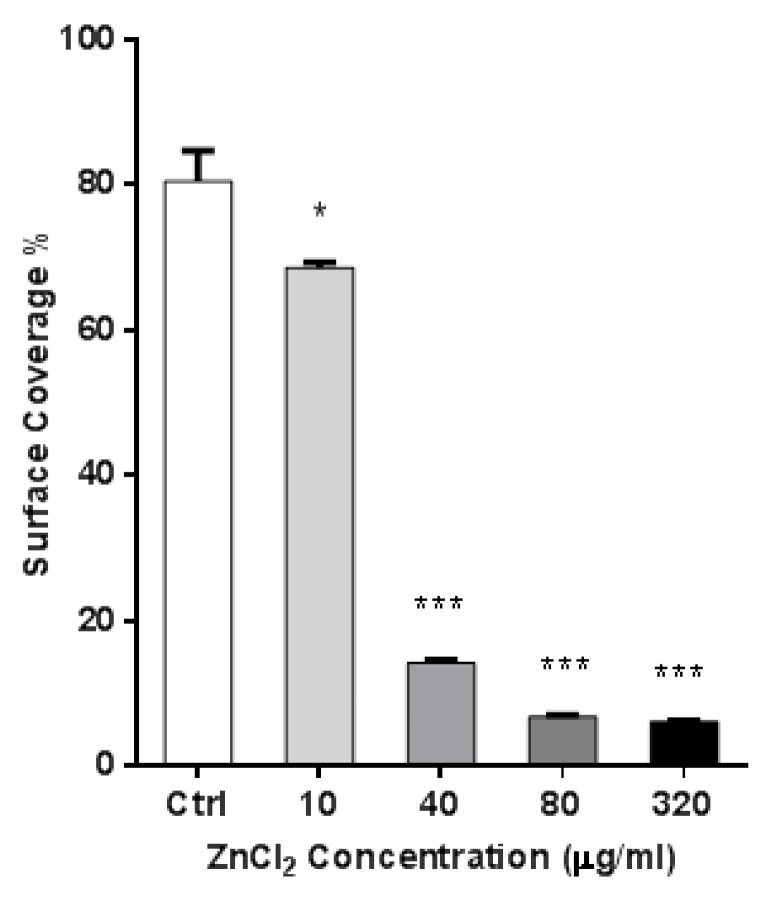
Stainless-steel surface coverage by A549 cells after ZnCl_2_ treatment, as revealed from SEM images. Asterisk presents significant difference with respect to the untreated control cells (* equals *p* < 0.05; *** equals *p* < 0.001; unpaired *t*-test).

As shown in Figure 6, an average of 81 ± 4 surface coverage was obtained for the control group. Surfaces covered by the A549 cells and treated with 10 µg/mL of ZnCl_2_ showed a reduction in cell coverage (70 ± 3) on the surface of stainless steel. From the concentration of 40 µg/mL, a significant decrease in surface coverage can be seen, and this trend gradually continued to the concentration of 320 µg/mL, where only 6 ± 1% of the surface was covered by cells. 

**Figure 7:**
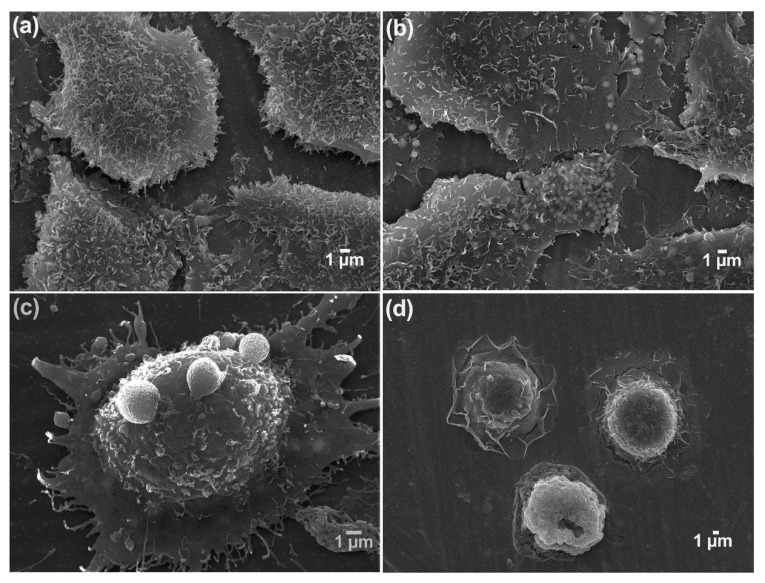
Morphology of A549 cells on stainless-steel surfaces after 48 h ZnCl_2_ treatment (SEM images): (**a**) control cells; (**b**–**d**) A549 cells treated with 10, 40, and 320 µg/mL of ZnCl_2_.

A change in morphology after the ZnCl_2_ treatment was also observed in the samples treated with different concentrations of ZnCl_2_. As depicted in Figure 7, the treated samples, especially at higher concentrations of ZnCl_2_, showed significantly different morphologies in comparison with the control. Visibly, the outer cell membranes of the non-treated cells in the control group were covered with microvilli, which can be interpreted as a higher cellular interaction with the surface and normal cell growth. Although most of the non-treated human lung A549 cells on the stainless-steel surfaces were highly flat and elongated with nearly smooth-edged and pleomorphic microvilli attached firmly to the cells, a gradual squeezing behavior was observed in the treated samples. At the lowest concentration of ZnCl_2_ (10 µg/mL), the cells were growing normally, but they gradually began to squeeze themselves with elevation of the ZnCl_2_ concentration. In the 40µg/mL-treated samples, the cell membrane became rough and perforated. Moreover, the cytoplasm loss, plasma membrane blebbing, and cell lysis were visualized. There were also apoptotic cells, which were detached from the surface and the neighboring cells. A few attached cells were observed in the highest concentration, but all were shrunken and circular.

### 3.5. Results of Electrochemical Cell-Based Impedance Spectroscopy (ECIS)

As the ECIS measurement progressed, it can be seen in the Nyquist plots (Figure S1) that there are certain differences between the tested samples and that there is a steadily increasing response. Certain anomalies were observed during the highest concentration (200 µg/mL), wherein the highest concentration has a slightly different response compared to the blank stainless-steel sample. In the Bode plot, the Z value remains relatively similar with minor differences, while the phase has certain changes. This anomaly can be seen at the highest concentration. The Nyquist diagrams are more or less straightforward; it can be observed that with the increasing concentration, the resistance parameter (Z’) drops, and the angle of the curve decreases. This, however, is not completely the case in the highest concentration, where the curve does decrease, but the resistance is close to concentration 40 μg/mL, and the capacitance factor (Z’’) is closer to the samples which are not treated with ZnCl_2_ (control).

**Figure 8:**
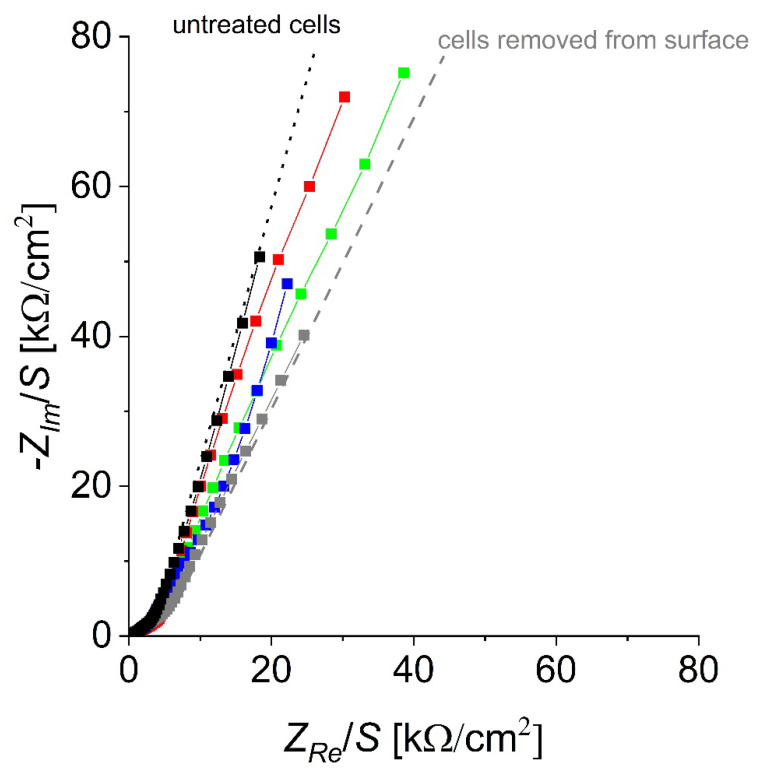
Nyquist diagrams of investigated biosensors responding to different ZnCl_2_ concentrations. Black label is the control and grey is the blank (positive control). Other concentrations presented: 9 μg/mL (red label), 40 μg/mL (green label), and 200 μg/mL (blue label).

During ECIS, the investigation focused on two areas: low and high frequency. In the low-frequency region (100–1 Hz), both the plain steel surface and the negative control have been shown to have lower Z values compared to the samples exposed to ZnCl_2_. This might be due to the potential corrosion activity of Cl- ions, which suggests that it would be difficult to determine a change in cell adhesion using solely the resistance measurement. In the higher frequency region (10–100 kHz), the Z values are lower, which suggests that the selected phosphate buffer has good conductivity; however, the Z values for the samples with 9 μg/mL and 40 μg/mL in this range are higher than both the negative control and the blank steel sample. Even at increased concentrations (200 μg/mL), the signal becomes distorted since it is possible that the Zn^2+^ ion solution at higher concentrations begins to interact with the steel surface. Nevertheless, the data can still be clearly interpreted as a valid response of the cells that are exposed to Zn^2+^ ions. The Nyquist diagrams are presented in Figure 8, while the Bode diagram is presented in the supporting information (Figure S1).

Much more can be seen when investigating the Bode phase values, where the high-frequency samples exposed to ZnCl_2_ have lower phase values (20–0°), and, in lower frequencies of 1–10 Hz, it can be seen that the phase is slowly decreasing. Here, it can also be seen that the responses vary between each sample. For the cell-covered samples, it can be seen that with an increased concentration of ZnCl_2_, the phase shifts to lower values. The phase angle data collected at 1 Hz were compared between the samples, and it was observed that over the course of the data, the angle increases when comparing an electrode with untreated cells (control) with an electrode without cells (blank). With the exception of the measurement at a low concentration (9 μg/mL), the data suggest that the phase angle’s increase at low frequencies may be a valid form of identification if the biological material has changed its properties. These data were compared to previously published measurements [38], in which measurements completed in combination with HepG2 liver cells show similar results with an increase in the phase angle at lower frequencies when exposed to different hepatotoxins.

The data presented in the table suggest that the concentration of 9 µg/mL of ZnCl_2_ is insufficient to reliably detect the effect of Zn^2+^ ions on cells, while the higher concentrations have negative phase angles much closer to the measurement, and the electrode had no present cells. In addition, it can be observed that a similar response can be observed in measurements performed with different types of cells and substances, in which the measurement with an added substance has a negative phase angle and a value quite close to that of the measurement taken with an electrode with no present cells. This is, of course, in accordance with the Nyquist diagrams, where it can be seen that with the increasing concentration, the plots seem to shift toward a measurement where no cells are present on the surface.

For the control group (which can be set as an example), it can be observed that the phase is significantly lower than the rest of the measurement. Finally, the measurement at the maximum concentration reveals that, at lower frequencies, the signal is changed compared to the lower concentration samples, which could be due to side reactions of the steel with the ZnCl_2_ salt. The Bode diagrams are shown in Figure 8, while the phase angle changes are shown in Table 1. The ECIS data correlate with other measurements, confirming that there are changes in the surface of the electrode with an increased ZnCl_2_ concentration [39].

## 4. Discussion

This study shows a proof of concept for a cell-based biosensor, composed of A549 cells attached to a stainless-steel surface, to electrically quantify the toxicity of metal ions in the liquid medium using low-frequency impedance spectroscopy. Our biosensor setup used steel foil electrodes that are produced on a large scale [25], thus ensuring their availability as an inexpensive electrode material [26]. We choose A549 cells that are able to form a monolayer of relatively evenly distributed cells that cover the stainless-steel surface (in comparison to other types of cells) [29]. The ZnCl_2_ solution was used as a test chemical in different concentrations to provoke toxicity. Conventional cytotoxicity methods and microscopy were used in parallel to confirm the information provided by the ECIS measurement.

The SEM microscopy revealed changes in the cell morphologies and the patterns of cell spreading and attaching to the surface on a nanoscale, even in the lowest Zn^2+^ ion exposure concentrations, in which there were no significant changes in the number of viable cells. However, at the highest Zn^2+^ ion exposure concentration, the surfaces were barely covered with cells, and the remaining cells were mostly dead and already detached. It can be seen that increasing the concentration of Zn^2+^ ions result in a significant change in the cell morphology, leading to the death and detachment of cells from the surface and, eventually, decreasing the surface coverage. This shrinkage and detachment of the dying cells from the electrodes was also detected as a change in the impedimetric signal.

The results were in line with the literature data, which describes that cell death on the electrode surfaces of the biosensors at cytotoxic concentrations changes the measured impedance [15,16,37,40,41]. The SEM revealed changes in the pattern of cell spreading, which is also detected as a significant change in the impedance magnitude in the special frequency range and pronounces a phase shift [16,37] and changes in the impedance signal [37,42]. An evident change in the ECIS signal for the groups exposed to Zn^2+^ ions was observed, compared to the control group, which confirms the effect of Zn^2+^ ions on the change in cell morphology and attachment [43]. The impedance spectroscopy data suggest that it is possible to detect the effect of Zn^2+^ ions in concentrations as low as 9 µg/mL of ZnCl_2_.

This study shows that the described stainless-steel A549 cell-based biosensor does not require a wide-frequency impedance measurement and can use only low-frequency ECIS instruments, which are usually less expensive than typical ECIS sensors. By focusing on a limited low-frequency range, the obtained data provide the same conclusions as the fully measured spectra. Although there are commercially available ECIS sensors that allow real-time cell analysis of adherent cells [44] and measure the cell impedance, reflecting cell growth, death, adhesion, and morphology [45,46], they are expensive and limited only to laboratory use [47,48]. An additional strength of the approach presented here is that it can detect the effects of ions and a mixture of ions and nano- or other particles, including their antagonistic or synergistic effects, which is not possible via other conventional approaches, such as in vitro cytotoxicity testing of individual substances.

## 5. Conclusions

A stainless-steel A549 cell-based biosensor has been successfully developed to detect the effect of Zn^2+^ ions as a reference testing chemical in a liquid medium using low-frequency impedance spectroscopy. The results showed that the presented biosensor can evaluate the cytotoxic response of cells, which makes it suitable for a broad range of sensing applications. The presented biosensor revealed a remarkable sensitivity to Zn^2+^ and has established repeatable, predictable, measurable responses between different ZnCl_2_ concentrations and impedance readings, along with microscopy observations. It was observed that the difference between the treated and control cells was clearly measurable. The cytotoxic effect of Zn^2+^ ions was observed as a significant resistance decrease. This impedance spectroscopy technique can be used to detect a signal in a low-frequency region or measure resistance in a direct current setting. The method presented here is an inexpensive way to detect the effects of different compounds and their mixtures as well as nano- or other particles, which are harmful to selected types of cells, and may point to their antagonistic or synergistic effects. It is, in particular, useful in detecting the effects of nanoparticles that dissolve. In such a case, both the dissolution dynamics and the concentration of ions are responsible for the effect. This would present an interesting and low-cost alternative to the already present commercial solutions for the detection of the hazardous potential of pollutants in a liquid medium before other more sophisticated methods are selected.

## Figures and Tables

**Table 1:** Comparison of negative phase angle change between electrode response with control group, treated group, and blank for all measurements. The values were taken at a frequency of 1 Hz.

Cell Type, Substance	Phase Angle for Untreated (- °)	Concentration-Phase Angle (- °)	Phase Angle for Cells Removed (- °)
A549, ZnCl_2_	66.801	9 µg/mL—67.065 40 µg/mL—62.15 200 µg/mL—59.493	56.45
HepG2, Aflatoxin B1 *	54.61	250 µM—47.67	46.911
HepG2, Isoniazid *	55.63	250 mM—50.927	52.378

* Marked data were obtained from one of the authors (Ref. [38]) and processed in the same way as the data obtained for this work.

## Data Availability

Data can be obtained from authors upon reasonable request.

## Supplementary Materials

The following supporting information can be downloaded at: https://www.mdpi.com/article/10.3390/nano12193493/s1. Figure S1: Bode diagram: A simultaneous representation of both total impedance (circles) and phase response (triangles) is shown. The color selection is the same as for Figure 7. The data for different concentrations of 9 μg/mL (red label) 40 μg/mL (green label), 200 μg/mL (blue label) are presented as well as for negative sample (black label) and blank stainless-steel sample that counts as the positive control (grey label); Table S1: Shows surface coverage and morphology of cells in the presence of a different concentration of ZnCl_2_ as a chemical using SEM.
