# Supplementary material for: A549 Cell-Covered Electrodes as a Sensing Element for Detection of Effects of Zn2+ Ions in a Solution
Source: Nanomaterials (Basel). 2022 Oct 6;12(19):3493. doi: 10.3390/nano12193493 (PMC9565818; doi:10.3390/nano12193493)
Supplement: Supplementary file 1 [file nanomaterials-12-03493-s001.zip › nanomaterials-1916088-supplementary.pdf]

Supplementary data:

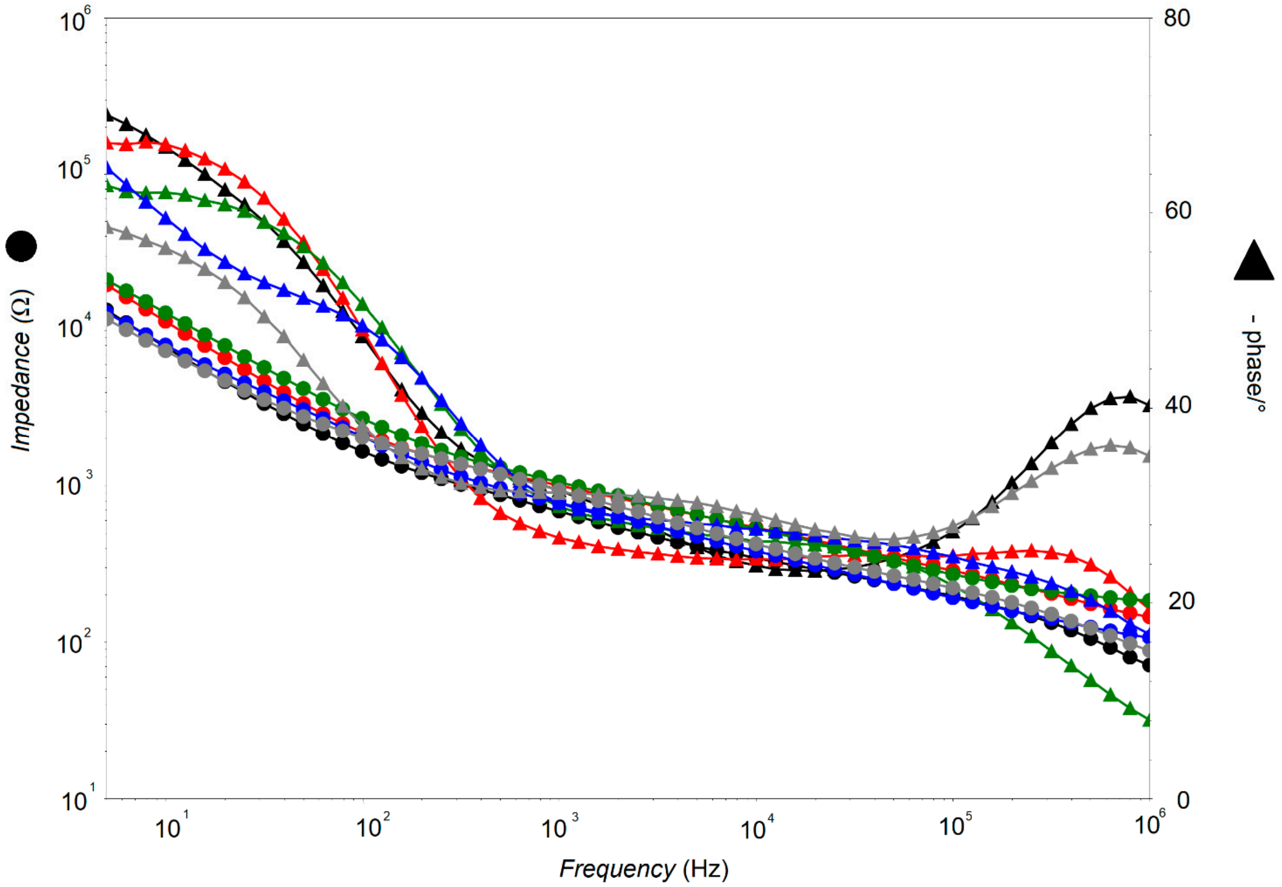

Figure S1. Bode diagram: A simultaneous representation of both total impedance (circles) and phase response (triangles) is shown. The color selection is the same as for Figure 7. The data for different concentrations of 9μg/mL (red label) 40 μg/mL (green label), 200 μg/mL (blue label) are presented as well as for negative sample (black label) and blank stainless-steel sample that counts as the positive control (grey label).

Table S1. Shows surface coverage and morphology of cells in the presence of a different concentration of $ZnCl_2$ as a chemical using SEM.

**The concentration of $ZnCl_2$ as a toxic compound**	**Surface Coverage(X100)**	**Shape of Cells(X500)**	**Single cell Example(X1000)**
Control	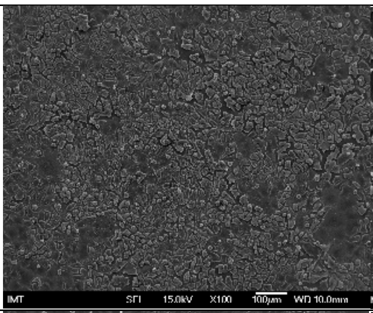	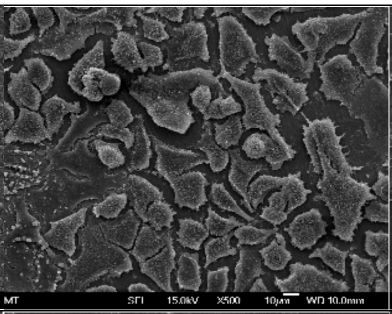	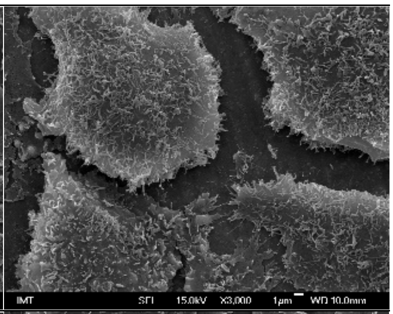
10 μg/mL $ZnCl_2$	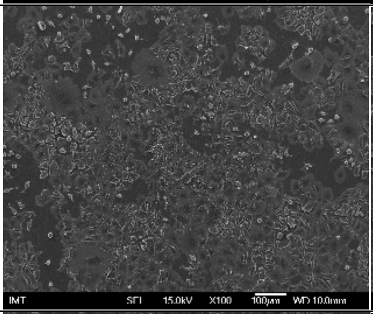	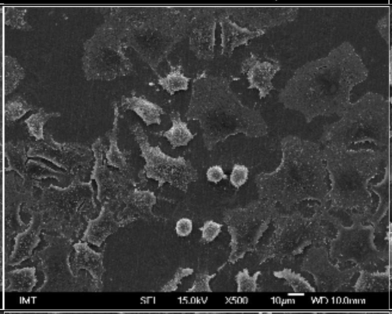	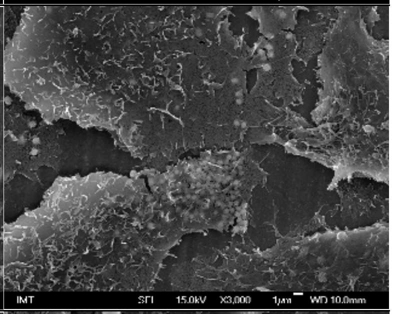
20 μg/mL $ZnCl_2$	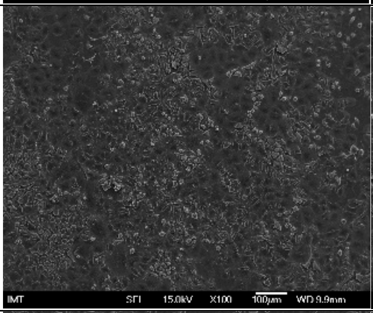	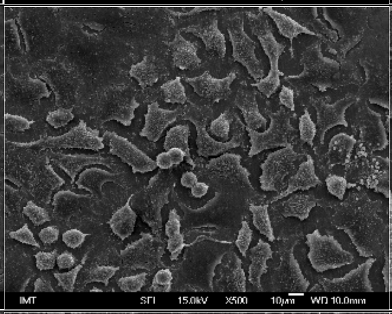	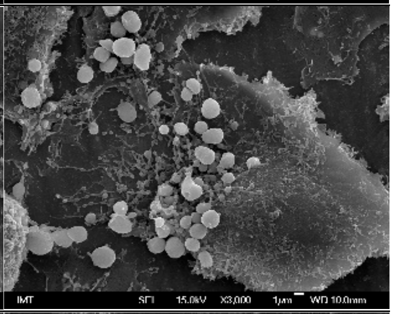
30 μg/mL $ZnCl_2$	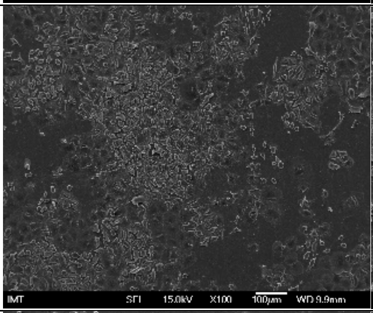	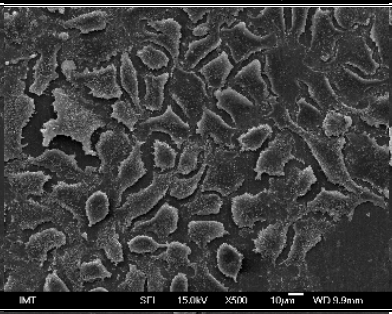	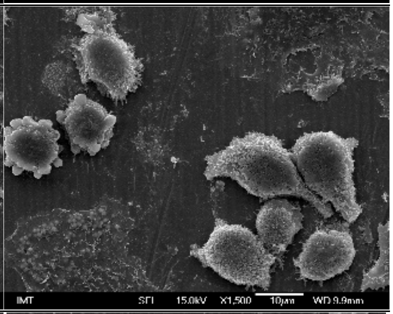
40 μg/mL $ZnCl_2$	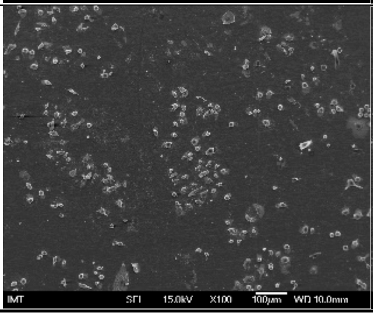	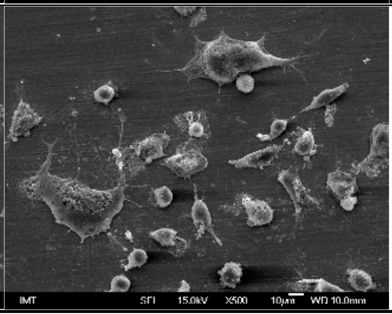	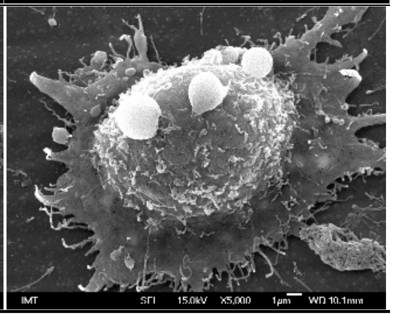

80 µg/mL $ZnCl_2$	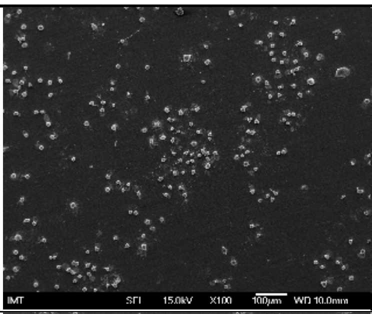	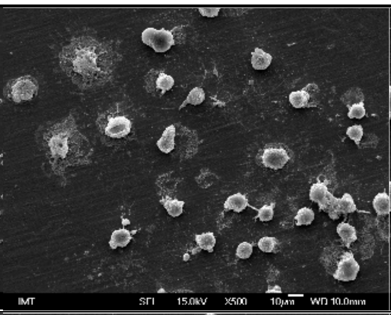	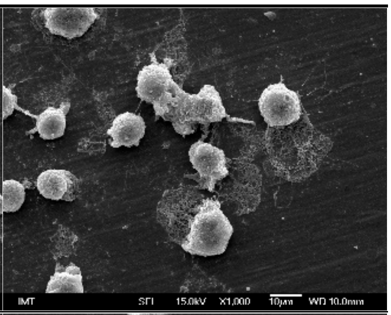
160 µg/mL $ZnCl_2$	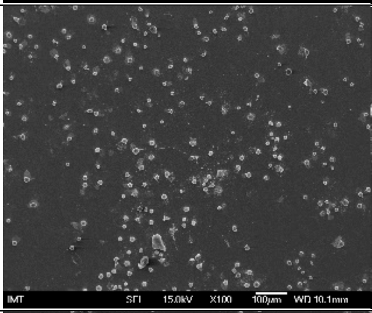	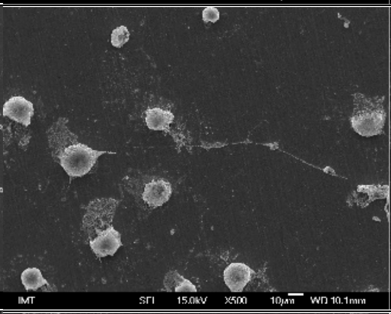	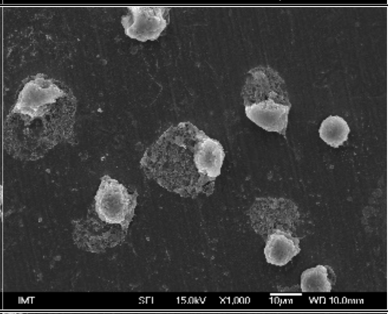
320 µg/mL $ZnCl_2$	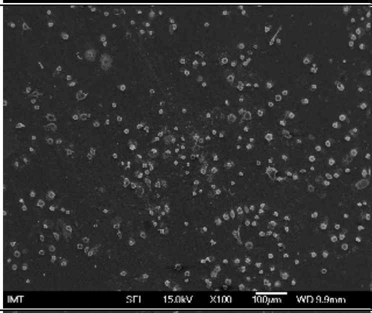	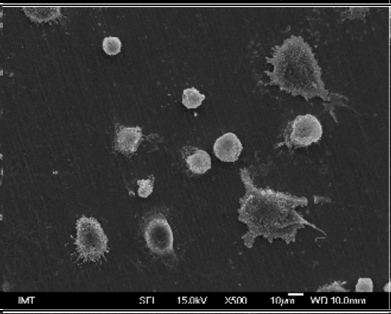	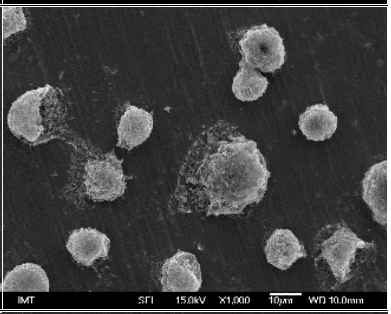
500 µg/mL $ZnCl_2$	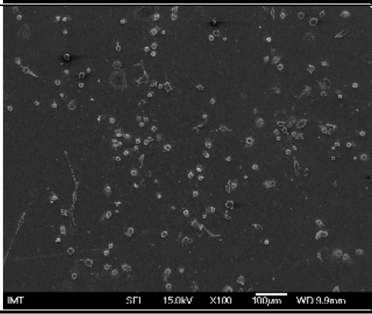	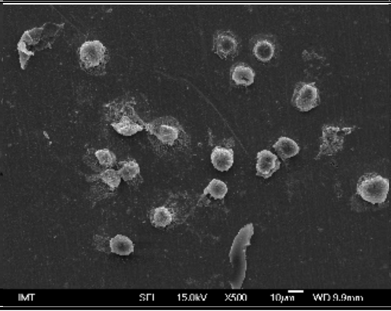	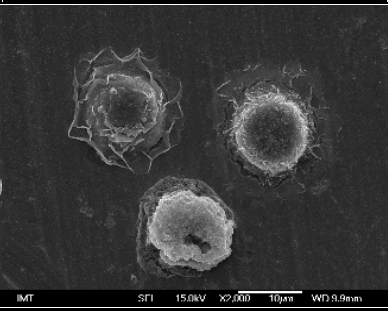
